# Optimizing efficiency and sustainability: ANN-controlled bi-directional EV battery charger with solar PV integration

**DOI:** 10.1038/s41598-026-46047-2

**Published:** 2026-03-26

**Authors:** Ramprasad Poojary, Renganathan Perumal, H. K. Sachidananda

**Affiliations:** 1 School of Engineering and IT, Manipal Academy of Higher Education, Dubai, United Arab Emirates; 2 Schindler Olayan Elevator Co. Ltd, Riyadh, Saudi Arabia

**Keywords:** Bidirectional EV charging, Artificial neural network control, Modified SEPIC converter, Solar photovoltaic integration, Grid-to-vehicle and vehicle-to-home systems, Renewable energy-based EV charging, Energy science and technology, Engineering

## Abstract

This study presents the design and performance evaluation of a bidirectional electric vehicle charging system integrating solar photovoltaic energy with an Artificial Neural Network based control strategy. The proposed architecture employs a modified Single-Ended Primary Inductor Converter capable of supporting both Grid-to-Vehicle and Vehicle-to-Home operating modes while maintaining stable bidirectional power flow between the grid, photovoltaic source, and EV battery. The ANN controller dynamically regulates the duty cycle of the MOSFET switches using battery current feedback and reference current signals, enabling adaptive control under varying solar irradiance and grid conditions. Simulation results indicate that the proposed system achieves charging efficiencies above 90% while maintaining stable operation for both 72 V and 240 V EV battery configurations. Compared with conventional proportional–integral control approaches, the ANN controller demonstrates faster transient response and improved current regulation during dynamic operating conditions. The integration of solar photovoltaic energy further reduces reliance on grid power and enhances renewable energy utilization in EV charging infrastructure. These results indicate that the proposed ANN-controlled bidirectional charging system provides an efficient and flexible solution for renewable-integrated EV charging applications.

## Introduction

From the recent literature review it is understood that significant progress in integrating Artificial Neural Networks (ANNs) with electric vehicle (EV) charging systems are used to improve efficiency, optimize power flow, and enhance sustainability. ANN-based controllers have consistently performed better as compared to conventional proportional-integral (PI) controllers in hybrid energy systems by regulating power distribution between batteries and ultra-capacitors^1^. Also, the Bidirectional chargers equipped with ANN control have shown improved grid voltage regulation during charging and discharging operations^2^. In addition to that, integrating photovoltaic (PV) systems with bidirectional chargers enables simultaneous PV energy conversion and battery charging/discharging while considering irradiance fluctuations and battery characteristics^3^. Energy management systems such as ANN-based Energy Management Systems (EMS) further strengthen such systems by reducing grid energy consumption by up to 28% in PV-powered charging stations with battery backup and V2G support^4^. All these developments lead to collectively highlighting the potential importance of ANN-driven control for achieving efficient, adaptive, and sustainable EV charging.

Rajalakshmi and Nisha^5^ has studied the importance of role of ANNs into broader smart grid applications. Also, ANNs support intelligent control of energy storage systems, enabling precise, real-time regulation of charging and discharging cycles in microgrid environments. The ANNs ability to respond adaptively to intermittent renewable resources, particularly solar and wind-makes them valuable for maintaining system stability under highly variable conditions. ANN-controlled bidirectional chargers also contribute to grid resilience by providing reactive power support during peak demand periods and by reducing total harmonic distortion (THD), thereby improving overall power quality^6^. These ANNs systems have also been successful in integrating with dual-stage converters in standalone PV systems to maximize power output and compensate for irradiance fluctuations, enhancing conversion efficiency and ensuring reliable operation^7,^^8^. These above studies clearly demonstrate the benefits of ANN-based controllers and highlight challenges related to ANN training complexity, data requirements, and real-time implementation constraints.

Apart from controller advancements, research in EV charging technologies emphasizes the need for rapid, grid-resilient infrastructure. In this context a comprehensive review by Sandeep and Anita^9^ examined the power quality and stability challenges associated with EV charging systems integrated with grid and photovoltaic energy sources. Their study highlighted that maintaining stable voltage and power quality is a critical requirement for large-scale EV adoption, particularly when renewable energy sources introduce variability in the charging infrastructure. The authors emphasized that continued research is required to develop improved control strategies capable of maintaining stable system operation under dynamic grid and generation conditions. These observations underline the need for intelligent control mechanisms in EV charging systems. In this context, the present study proposes an ANN-based control strategy for a bidirectional EV charger to enhance system stability, improve charging performance, and support effective integration of solar PV energy within the charging infrastructure^10^.

The recent evolution of EV infrastructure, including standardized connectors and intelligent control strategies, is essential for improving user experience and charging performance^11^. Bidirectional V2G systems further enhance grid stability by enabling EVs to supply energy back to the grid, balancing supply-demand mismatches and supporting renewable energy penetration^12^. Along with these advanced control strategies such as model predictive control and adaptive virtual synchronous generator control will ensure stable and seamless bidirectional energy transfer^13^. Despite these technologies, issues of cost, interoperability, and grid stability continue to present major barriers that future research must address.

Beyond EV-specific applications, ANNs have been widely implemented across multiple energy sectors to optimize system performance and reduce energy consumption. Their ability to process large datasets and learn non-linear patterns enables accurate prediction of energy demand influenced by environmental conditions, occupancy, and operational behavior^14^. Case-study evidence as shown by Martina et al.^15^ shows substantial savings when neural networks are used for machine-level optimization, such as a 50,000-kWh annual reduction achieved in a plastic manufacturing SME. The study by Sanjeeb et al.^16^ using functional optimization using Neural Networks (FONN) approach further demonstrates the potential of ANNs in minimizing energy functional at discrete interaction points, improving efficiency across complex engineering processes. ANNs have also been effectively used to model converter efficiencies in telecom networks^17^ and to improve building energy management using optimized feed-forward architectures^18^.

The integration of Solar PV-EV in sustainable transportation represents an important pathway and solar-powered EV charging reduces energy costs^19,20^, mitigates voltage drops during peak charging^21^, and decreases greenhouse gas emissions by reducing dependence on fossil fuels^22^. Building-integrated PV (BIPV) models additionally enable structural energy generation, improving space utilization and promoting energy circularity^19^. Also, Advanced optimization algorithms further enhance PV-battery coordination, reducing grid stress and maximizing renewable utilization^22^. But challenges in widespread implementation include high installation costs, infrastructure limitations and variability in irradiance.

Sandeep and anita^23^ have reviewed power quality and stability of EV charging with grid and PV solar and considered it is essential for widespread adoption since these systems have been improved based on generation and demands of the power grid. They concluded that continued research and development is necessary to upgrade the power quality.

At the grid level, bidirectional power flow introduces both challenges and opportunities. Increased EV penetration can destabilize power systems, especially in hybrid microgrids where AC-DC interactions may trigger frequency deviations and negative capacitance effects^22,24^. Microgrids capable of independent operation offer enhanced resilience, especially during outages^25^. Load modelling research further reveals that traditional constant power models inaccurately represent EV charging behavior, while ZIP models incorporating state-of-charge-dependent voltage characteristics provide more realistic assessments of system losses and voltage profiles^26^.

Sandeep and anita^27^ have studied optimized energy management in grid connected solar PV battery for enhancing the stability and power quality. They used the honey badger optimization algorithm for energy management in battery connected solar PV. They implement this proposed work in MATLAB/Simulink tool, and the results are evaluated. They concluded that the proposed method improved the power quality for voltage and current respectively. Indrajit and Provas^28^ have studied optimized design and hybrid photovoltaic (PV), wind turbine (WT) distributed generation system and battery energy storage system using dynamic arithmetic optimization algorithm (DAOA) which is commonly used in electrical engineering to solve optimization tasks. They concluded that DAOA is superior to several optimization methods. Sunanda et al.^29^ has studied power grid incorporating electric vehicles (EVs) to check its significant impact in safe and reliable operation. But if it applied to wind power then there is an unpredictable nature. So, they combined wind energy and solar energy as wind is always available as compared to solar energy. So, they concluded that renewable energy sources (RESs) perform better as compared to conventional systems. Sourav et al.^30^ has studied renewable energy sources (RESs) considering wind, solar photovoltaic (PV)and hydro to find the optimal design using hummingbird algorithm. They used the probabilistic optimal power flow (POPF) to address the inherent uncertainties related to power output of RESs. They concluded that POPF system has superior over sophisticated modern approaches. Chandan et al.^31^ has studied optimal power flow (OPF) of the combined heat and power economic dispatch (CHPED) problem integrated with renewable sources and using a new practical approach using chaotic oppositional sine cosine algorithm (SCA) (COSCA). They combined renewable energy sources such as wind-solar-EVs and integrated with the system for environmental sustainability. They concluded that the suggested COSCA algorithm has shown well established optimization techniques. Recent research highlights the increasing importance of integrating renewable energy resources and electric vehicles within modern power systems to enhance energy efficiency and grid stability. For example, studies on optimal power flow and economic dispatch frameworks demonstrate that combining wind, solar, and EV resources can reduce fuel costs, emissions, and transmission losses while improving voltage stability and system reliability through advanced optimization algorithms^32^. Similarly, optimization-based approaches such as whale optimization and other metaheuristic techniques have been applied to complex energy scheduling problems to improve convergence speed and solution quality in multi-objective power system optimization^33^. In addition, recent work on renewable-integrated grid systems emphasizes the role of intelligent optimization strategies in maximizing renewable energy utilization and managing the nonlinear constraints associated with economic load dispatch and grid operation^34^.

The increasing penetration of renewable energy resources has become a key strategy for addressing global energy sustainability, reducing greenhouse gas emissions, and improving the resilience of modern power systems. Renewable technologies such as solar photovoltaic and wind energy provide clean and sustainable electricity generation, while their integration with advanced power electronic converters and intelligent control strategies enables improved energy efficiency and grid flexibility. Recent studies have shown that renewable-integrated power systems can enhance system stability, reduce dependency on fossil fuels, and support decentralized energy management within smart grids and electrified transportation infrastructures^35–39^. In particular, the integration of renewable energy with electric vehicle charging infrastructure has attracted increasing research attention because EVs can act not only as transportation devices but also as distributed energy resources that support grid balancing and renewable energy utilization. At the same time, battery technologies play a crucial role in enabling efficient energy storage, power balancing, and bidirectional energy exchange in renewable-integrated systems. Advances in battery thermal management, energy storage optimization, and lifecycle performance have significantly improved the operational reliability and energy density of modern electrochemical storage systems^40–43^. These developments highlight the importance of integrating renewable generation, intelligent control strategies, and advanced battery storage technologies in the design of next-generation EV charging architectures.

Sustainability has become a central objective in modern energy systems due to the need to reduce carbon emissions, enhance energy efficiency, and support reliable integration of renewable energy resources. Recent studies highlight that intelligent management of distributed energy resources and smart grid technologies can significantly improve demand response, system reliability, and overall sustainability of power networks, particularly when renewable generation and electric mobility are integrated into grid operations. In this context, advanced optimization and control strategies are increasingly required to maintain stable power system operation while maximizing renewable energy utilization and reducing environmental impact. Artificial Neural Networks (ANNs) have emerged as powerful tools for such applications because they can learn nonlinear relationships, adapt to varying operating conditions, and provide accurate prediction and control capabilities in complex energy systems. Consequently, ANN-based approaches are widely used in power electronics, energy management, and intelligent control frameworks to enhance system performance, efficiency, and decision-making in renewable-integrated energy infrastructures^44–50^.

Existing studies on EV charging systems have often examined intelligent control strategies and renewable energy integration as separate research streams (Refer Table 1). Several studies have applied Artificial Neural Network controllers to regulate charging current and enhance converter performance in EV charging systems. However, most of these implementations are designed primarily for grid-connected charging environments and do not explicitly prioritize renewable energy utilization. In parallel, photovoltaic-assisted EV charging systems have been proposed to reduce dependence on conventional grid electricity. Nevertheless, many of these systems rely on conventional control techniques such as proportional–integral controllers or rule-based energy management approaches, which may exhibit limited adaptability under rapidly changing solar irradiance and load conditions.

**Table 1:** Comparative positioning of the proposed ANN-controlled bidirectional EV charger relative to recent research (based on the cited studies).

References	System/topology	Control strategy	Renewable energy integration	Bidirectional operation	Key focus	Limitation compared with proposed work
Rakesh et al.^2^	Bidirectional EV battery charger	ANN-based control	Not considered	Yes	Robust charging control using ANN	Does not include solar PV integration
Choi et al.^3^	Bidirectional EV charger with PV PCS	Conventional control	Solar PV integrated	Yes	PV-assisted EV charging architecture	Does not apply intelligent ANN control
Soumya et al.^4^	PV-powered EV charging station with battery backup	ANN-based energy management	Solar PV integrated	V2G supported	Energy management of PV-powered charging station	Converter-level ANN control for bidirectional charger not emphasized
Jyothsna et al.^7^	Grid-based EV charging system	ANN controller	Not considered	Not specified	ANN-based charging control	Renewable integration and bidirectional energy exchange not addressed
Izviye and Erdal^12^	Bidirectional EV fast charging station in DC microgrid	Model Predictive Control	Microgrid integration	Yes	Predictive control for fast charging	Does not use ANN-based adaptive control
Utsav and Bhim^51^	Bidirectional EV battery charger	Conventional control	Not considered	Yes	Bidirectional charging for EV batteries	Does not include renewable PV integration or ANN control
Proposed work	Modified bidirectional SEPIC EV charger	ANN-based adaptive control	Solar PV integrated	G2V and V2H supported	Renewable-integrated intelligent EV charging	Simulation validation, hardware implementation planned in future work

Despite these advancements, only limited research has investigated the integration of intelligent control with renewable energy management within a unified bidirectional EV charging architecture. Many existing systems either focus on ANN-based control for battery charging processes or on photovoltaic-powered EV charging stations without incorporating adaptive control mechanisms for bidirectional power exchange. As a result, the interaction between renewable energy variability, EV battery dynamics, and bidirectional energy exchange with residential loads remains insufficiently addressed.

Furthermore, several technical challenges remain in the development of efficient EV charging infrastructures. First, conventional EV charging systems frequently rely on proportional–integral control strategies that may not perform effectively under nonlinear and time-varying operating conditions introduced by solar photovoltaic generation and grid voltage fluctuations. Second, many charging architectures are designed for unidirectional energy transfer, limiting their ability to support advanced functionalities such as Vehicle-to-Home or Vehicle-to-Grid energy exchange. Third, the integration of renewable energy sources into EV charging systems is often constrained by the lack of intelligent control mechanisms capable of maintaining stable power flow and power quality under dynamic environmental conditions. These limitations highlight the need for improved control strategies and converter architectures that enhance system adaptability, efficiency, and renewable energy utilization.

To address these research gaps, the present study makes the following contributions:

1. A bidirectional EV charging architecture is developed that integrates solar photovoltaic generation with grid-connected charging infrastructure, enabling both Grid-to-Vehicle and Vehicle-to-Home operating modes.
2. A modified SEPIC converter topology is introduced to facilitate stable bidirectional power transfer between the photovoltaic source, grid, and EV battery.
3. An Artificial Neural Network based control strategy is proposed to regulate the duty cycle of the MOSFET switches, enabling adaptive control under varying solar irradiance and grid operating conditions.
4. The proposed system supports multiple EV battery configurations, including 72 V and 240 V battery systems, demonstrating flexibility for different electric vehicle applications.
5. The performance of the proposed architecture is evaluated through simulation analysis, demonstrating improved charging stability, efficient power transfer, and enhanced renewable energy utilization.

## Methodology

### System architecture

**Fig. 1:**
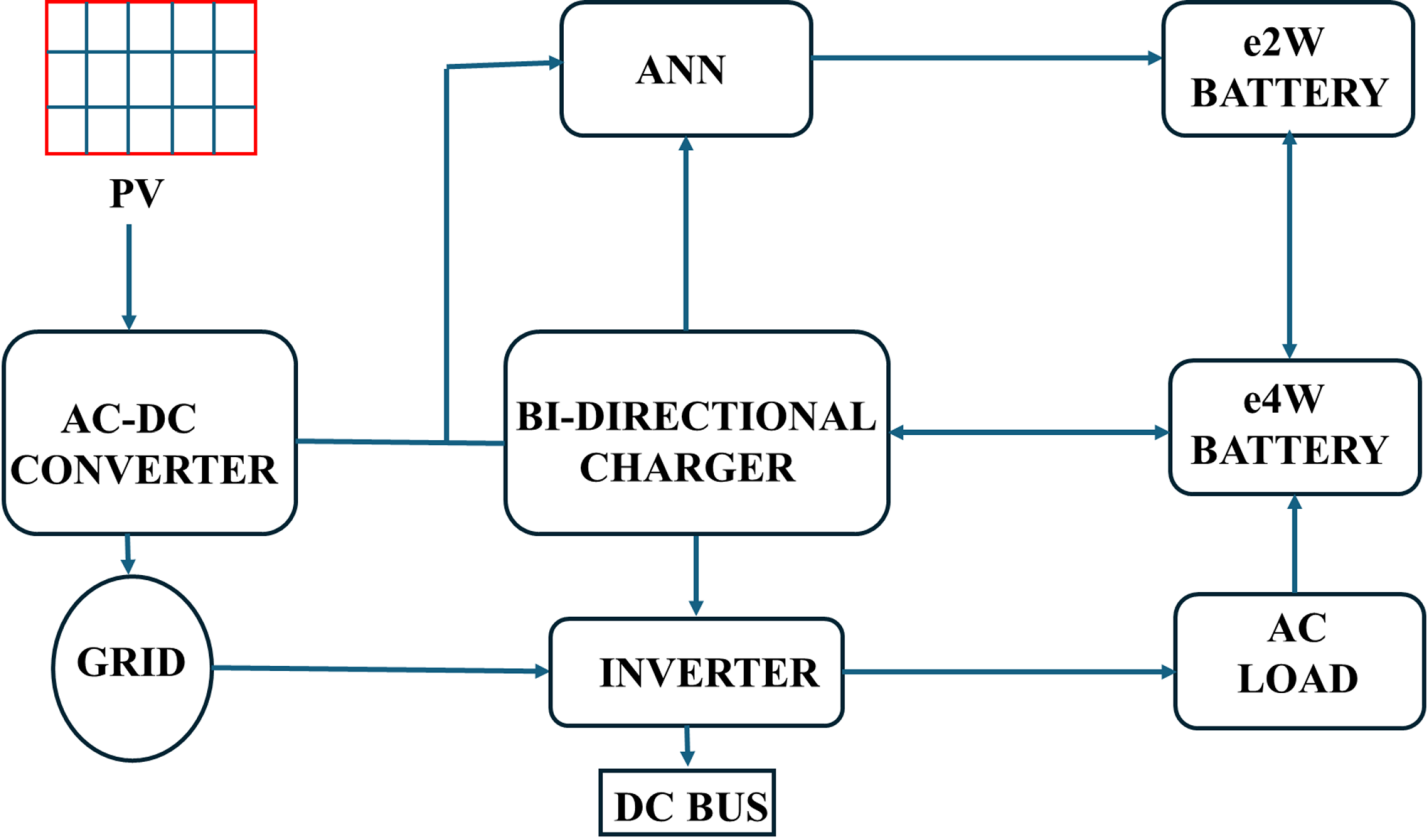
The system architecture of the ANN-controlled bi-directional EV charging system.

The proposed system integrates a bidirectional battery charger for electric vehicles (EVs) with solar photovoltaic (PV) input, grid interaction, and an Artificial Neural Network (ANN)-based control scheme (refer Fig. 1). The architecture supports both Grid-to-Vehicle (G2V) and Vehicle-to-Home (V2H) modes and is composed of four major subsystems:

- EV Charging Unit: It enables controlled bidirectional energy flow between the EV battery, grid, and solar PV system in turn supports G2V and V2H functionality.
- Solar PV System: It generates renewable energy and supplies it to the charger during G2V operation, in turn helps to reduce reliance on the grid.
- Grid Interface: Supports power draw from the grid during low PV availability and enables controlled V2H power injection.
- ANN-Based Control Unit: Maintains charging and discharging operations by adjusting MOSFET duty cycles in real time based on battery current, reference current, and operating mode.

A modified Single-Ended Primary Inductor Converter (SEPIC) forms the core power-processing stage, offering intrinsic bidirectional capability when configured with MOSFET switches.

### Significance for replacing PI with ANN

The existing PI controllers perform satisfactorily for linear, steady-state operating conditions and require manual tuning and exhibit limited adaptability under various nonlinear and time-varying scenarios such as irradiance fluctuations, battery ageing, and grid disturbances.

In contrast, the ANN:

- Adapts nonlinear relationships without explicit modelling.
- Adjusts control decisions dynamically.
- Ensures maintain stable current/voltage even under rapidly changing conditions.
- Helps to improve power quality by reducing waveform distortion.
- Enhances G2V and V2H performance through optimized duty-cycle generation.

This adaptability makes ANN more suitable for real-time EV charging applications involving renewable energy and bidirectional operation. The solar PV subsystem used consists of four parallel strings, each with five series-connected modules. Irradiance (1000 W/m^2^) and cell temperature (25 °C) were used as standard test conditions for simulation. Real-time PV output was continuously monitored and supplied to the SEPIC converter.

The ANN-managed controller prioritizes the use of solar energy during G2V operation and automatically switches to grid power during low irradiance, ensuring uninterrupted charger functionality.

### Solar PV model and energy management

The solar PV subsystem consists of four parallel strings, each with five series-connected modules. Standard test conditions (irradiance of 1000 W/m^2^ and temperature of 25 °C) were used. Real-time PV output was supplied to the SEPIC converter.

The ANN-managed controller prioritizes solar energy during G2V operation and automatically transitions to grid power during low irradiance, ensuring uninterrupted charger functionality.

### Modified SEPIC converter

The modified SEPIC converter enables regulated and isolated bidirectional power flow. Important design features include:

- **Bidirectionality**: It is achieved by replacing the conventional SEPIC diode with an actively controlled MOSFET.
- **Galvanic Isolation**: It ensures using a high-frequency transformer for safety during grid interaction.
- **Single-Stage Energy Conversion**: Helps to reduce switching losses compared to dual-stage architecture.
- **Wide Voltage Compatibility**: It helps to support both 72 V (two-wheeler) and 240 V (four-wheeler) EV batteries.
- **DCM Operation**: Facilitates near-unity power factor and reduces harmonic content in the supply current.

During G2V operation, the converter maintains constant battery current, and during V2H, it regulates the sinusoidal voltage at the point of common coupling.

In a conventional SEPIC converter topology, a diode is typically used as the rectifying element to allow energy transfer from the input source to the output load. This configuration restricts power flow to a single direction and therefore cannot support bidirectional energy exchange. Such unidirectional operation is suitable for traditional DC–DC power conversion but is insufficient for EV charging systems that require both charging and discharging capabilities.

To enable bidirectional power flow, the proposed system replaces the conventional diode with an actively controlled MOSFET switch. This modification allows the converter to operate in two modes depending on the switching strategy. During Grid-to-Vehicle operation, the converter functions similarly to a standard SEPIC converter, transferring energy from the grid or solar PV source to the EV battery. During Vehicle-to-Home operation, the switching sequence reverses the direction of energy transfer, allowing stored battery energy to supply the AC load through the inverter stage. This modification transforms the SEPIC converter into a bidirectional power processing unit capable of supporting both charging and discharging functions within the same converter structure. Compared with conventional unidirectional SEPIC designs, the proposed topology improves system flexibility and enables seamless integration of EV batteries as distributed energy storage resources within residential or grid-connected energy systems (refer Table 2).

**Table 2:** Structural and functional comparison between conventional SEPIC converter and the proposed modified SEPIC converter used in the bidirectional EV charging system.

Feature	Conventional SEPIC converter	Proposed modified SEPIC converter
Rectifying element	Diode	MOSFET switch
Power flow direction	Unidirectional	Bidirectional
EV charging capability	Only G2V	G2V and V2H
Control method	Passive rectification	Active switching control
Application	DC voltage regulation	Bidirectional EV energy management

### High-gain DC-DC converter design

**Fig. 2:**
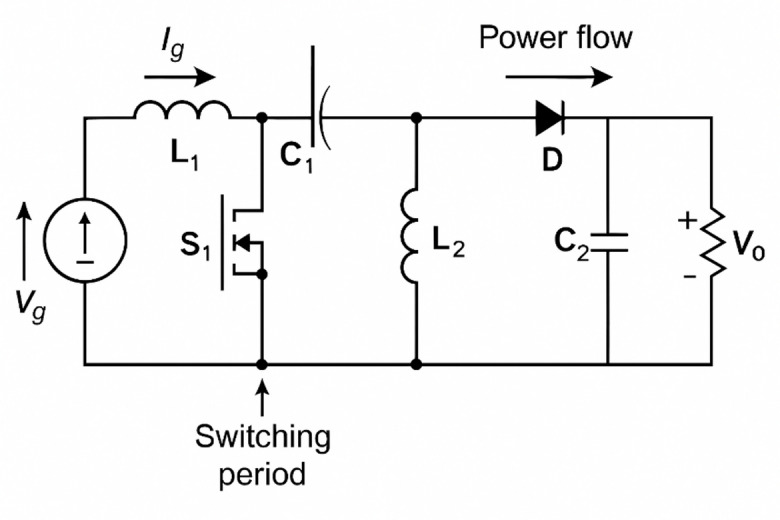
Schematic representation of the high-gain DC-DC converter topology integrating an input source V_g_, primary inductor L_1_, switching device S_1_, intermediate capacitor C_1_, secondary inductor L_2_, rectifying diode D, and output filter capacitor C_2_, supplying the regulated output voltage V_0_. The diagram illustrates the power transfer path and switching operation, where the controlled switching of S_1_ enables energy storage and release across the inductors and capacitors, achieving voltage boosting under varying load and input conditions. This converter configuration is suitable for electric vehicle charging systems and renewable energy interfaces requiring efficient, stable, and high step-up DC conversion.

A high-gain DC-DC converter was employed to regulate voltage between the solar PV subsystem and the DC bus of the proposed EV energy management system. This converter helps to provide the required voltage boosting capability under different irradiance and load conditions, ensuring stable charging performance for both two-wheeler (72 V) and four-wheeler (240 V) battery configurations. This design includes inductive energy storage, active switching control, and intermediate capacitive buffering to achieve high step-up ratios while maintaining low ripple characteristics. The schematic of converter topology is presented in Fig. 2.

### MOSFET switching and PWM control and ANN duty-cycle control

Two MOSFET switches manage directional power flow. A Pulse Width Modulation (PWM) generator produces high-frequency switching pulses whose duty cycle determines the charging or discharging rate.

The ANN provides the real-time duty-cycle command to the PWM system, enabling:

- Fast transient response.
- Reduced switching losses.
- Optimal power transfer under variable load and grid conditions.

**Fig. 3:**
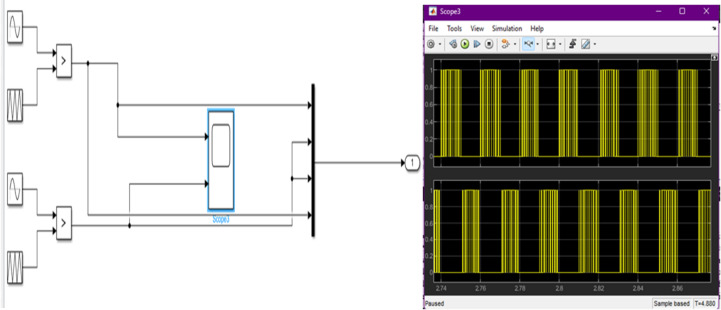
Simulink PWM generation model and corresponding switching waveforms. The left portion shows the Simulink block diagram used to generate the PWM control signals, including sinusoidal and carrier waveform inputs, comparators, and signal routing to the switching block. The right portion displays the resulting PWM pulses captured in Scope3, showing the modulation pattern and switching behavior over the sampled interval. This figure provides an integrated view of both the control logic and the generated gate pulses used for converter switching.

The PWM subsystem receives the duty-cycle input generated by the ANN controller. The Simulink model shown in Fig. 3 illustrates the comparator-based PWM generation mechanism, where sinusoidal reference and high-frequency triangular waveforms produce the switching pulses supplied to the MOSFETs. The resulting PWM waveform behavior is shown in the combined scope traces.

### ANN architecture and control strategy

The ANN serves as the primary controller for regulating G2V and V2H operations.

#### Control coordination and mode transition strategy

The proposed EV charging architecture integrates three primary subsystems: the solar photovoltaic energy source, the bidirectional charging converter, and the grid interface. Effective coordination among these subsystems is essential for maintaining stable system operation and enabling seamless transitions between charging and discharging modes. The control framework therefore employs an ANN-based supervisory mechanism that continuously monitors system variables and determines the appropriate operating mode.

During Grid-to-Vehicle operation, the EV battery is charged using energy supplied by the solar PV array and the utility grid. The control algorithm prioritizes the use of photovoltaic energy whenever available. When PV generation exceeds the charging demand, the ANN regulates the converter duty cycle to maintain the desired charging current while minimizing grid power consumption. If solar irradiance decreases and PV output becomes insufficient, the controller automatically increases grid contribution to maintain the target charging profile. This adaptive behaviour ensures uninterrupted charging while maximizing renewable energy utilization. Vehicle-to-Home operation is activated when the system detects a demand from the connected AC load and the EV battery state of charge remains above the minimum allowable threshold. In this mode, the bidirectional SEPIC converter reverses the direction of power flow and delivers energy from the battery to the load through the inverter stage. The ANN controller regulates discharge current by continuously comparing the measured battery current with the reference value, thereby maintaining stable output voltage at the load interface.

The transition between operating modes is governed by a supervisory control logic based on system conditions such as battery state of charge, load demand, PV availability, and grid status. When the battery reaches the desired charging level or when external load demand increases, the control system shifts from G2V to V2H operation by adjusting switching signals and converter duty cycles. Conversely, when battery charge decreases below the predefined threshold or grid support becomes necessary, the system transitions back to G2V mode. This coordinated control strategy enables smooth and stable energy exchange among the PV system, EV battery, and grid infrastructure.

**Mode selection conditions**.

G2V mode activated when.

- Battery SOC below charging threshold.
- PV or grid energy available.

V2H mode activated when.

- Residential load demand detected.
- Battery SOC above discharge threshold.

Conventional proportional–integral controllers are commonly used in DC–DC converter applications because of their simple structure and straightforward implementation. However, their performance is highly dependent on fixed parameter tuning and accurate system modelling. In renewable-integrated EV charging systems, operating conditions frequently change due to variations in solar irradiance, grid voltage fluctuations, and dynamic battery characteristics. These nonlinear and time-varying conditions can reduce the effectiveness of PI controllers, resulting in slower dynamic response and reduced stability.

Artificial Neural Network controllers provide an alternative approach capable of handling nonlinear system behaviour. ANN models can learn complex relationships between input variables and control actions during the training process, allowing the controller to adapt to varying operating conditions without requiring precise mathematical modelling of the system. In the proposed charging architecture, the ANN controller regulates the converter duty cycle based on real-time current measurements, enabling improved dynamic response and more stable operation during both Grid-to-Vehicle and Vehicle-to-Home modes.

The Artificial Neural Network (ANN) controller dynamically regulates the switching duty cycle of the MOSFET devices in the bidirectional SEPIC converter based on real-time operating conditions. The ANN receives system measurements such as battery voltage, charging current, and reference current as input variables. During the training stage, the network learns the nonlinear relationship between these input parameters and the optimal duty cycle required to maintain the desired charging performance. During real-time operation, the trained ANN processes the instantaneous system measurements and generates an appropriate duty cycle command for the converter switches. This enables the controller to continuously adapt to variations in solar irradiance, grid voltage fluctuations, and changes in battery state of charge. As a result, the ANN-based controller provides adaptive current regulation and improved dynamic response compared with conventional proportional-integral controllers, which rely on fixed gain parameters and may exhibit reduced performance under nonlinear and time-varying operating conditions.

**Fig. 4:**
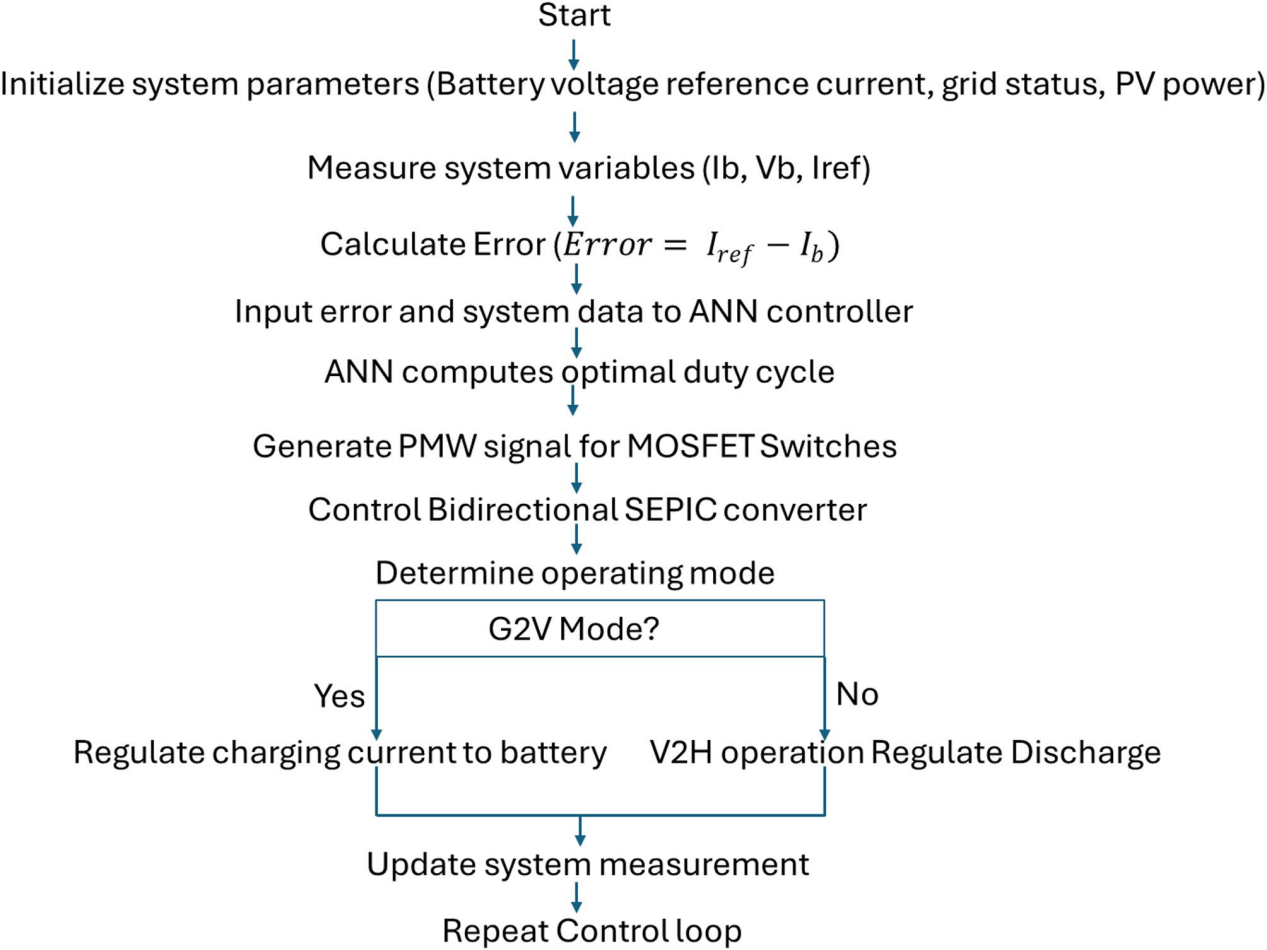
Flowchart illustrates the operational procedure of the ANN-based control strategy used for regulating the bidirectional EV charging system during Grid-to-Vehicle and Vehicle-to-Home modes.

The flowchart summarizes the closed-loop control procedure of the proposed ANN-controlled bidirectional EV charging system. Real-time measurements of battery current and the reference current are used to compute an error signal, which is processed by the ANN to generate the optimal MOSFET duty cycle and corresponding PWM signals. Based on the selected operating mode (G2V or V2H), the controller regulates charging or discharging current and continuously updates measurements to maintain stable bidirectional power flow (refer Fig. 4).

#### ANN structure

- Input Layer:
  - Measured battery current (Ib).
  - Reference current (Iref).
- Hidden Layers:
  - Two hidden layers.
  - 10 neurons each.
  - Sigmoid activation functions for nonlinear mapping.
- Output Layer:
  - Generates duty cycle (D).
  - Linear activation to produce continuous control values.

The selected neural network architecture consists of two hidden layers with ten neurons each. This configuration was chosen to provide sufficient representational capacity for modelling the nonlinear dynamics of the EV charging system while maintaining computational efficiency suitable for real-time control applications. Sigmoid activation functions were used in the hidden layers to capture nonlinear relationships between input variables, while a linear activation function was applied at the output layer to generate continuous duty-cycle values for PWM control. Preliminary simulations indicated that increasing the number of neurons beyond this configuration did not produce significant performance improvement while increasing computational complexity.

In the proposed system, the duty cycle of the MOSFET switch in the modified SEPIC converter is determined using an ANN-based control strategy. The controller continuously monitors the converter output current and compares it with the reference current required for the desired operating mode. The resulting error signal is used as an input to the ANN controller, which processes the system state and generates an appropriate control signal corresponding to the required duty cycle. This duty cycle is then used to generate pulse width modulation signals that drive the MOSFET switch of the converter. By dynamically adjusting the switching duty cycle in response to real-time system conditions, the ANN controller ensures stable current regulation and efficient power transfer during both Grid-to-Vehicle and Vehicle-to-Home operating modes.

#### ANN operation

The Artificial Neural Network (ANN) controller determines the optimal duty cycle of the converter switches based on the current regulation error. The error signal is defined as the difference between the reference current and the measured battery current, which represents the deviation that the controller must minimize. This formulation is commonly adopted in feedback control systems used in electronic power converters.

The ANN determines the optimal duty cycle based on the Error signal generated as^52^:

1 $$\:Error=\:{I}_{ref}-{I}_{b}$$

where $\:{I}_{ref}$represents the reference charging current and $\:{I}_{b}$denotes the measured battery current.

During Grid-to-Vehicle (G2V) operation, the ANN increases the converter duty cycle when the battery current is lower than the reference current and gradually reduces the duty cycle as the battery current approaches the desired reference value. During Vehicle-to-Home (V2H) operation, the ANN regulates the discharge current while maintaining stable voltage at the point of common coupling.

#### Training and optimization

The ANN model is trained using datasets obtained from simulated G2V and V2H operating conditions. The training process uses the backpropagation learning algorithm with gradient descent optimization to update network weights and minimize prediction error. The performance of the ANN during training is evaluated using the Mean Squared Error (MSE) loss function, which measures the squared difference between the reference current and the predicted current.

- **Training Data**: Collected from simulated G2V and V2H operations.
- **Learning Algorithm**: Backpropagation with gradient descent.
- **Loss Function**: Mean Squared Error (MSE).

The MSE loss function penalizes larger deviations between reference and measured current, enabling the ANN to converge toward accurate current regulation^53^.

2 $$\:MSE=\:\frac{1}{n}\sum\:{({I}_{ref}-{I}_{b})}^{2}$$

where $\:n$ represents the number of training samples.

##### Hyperparameters

Learning rate, epochs, and batch size are fine-tuned via cross-validation.

The trained ANN consistently generates faster, more accurate control decisions compared to fixed-gain PI controllers.

The training dataset used for the ANN controller was generated from dynamic simulations of the EV charging system operating under both Grid-to-Vehicle (G2V) and Vehicle-to-Home (V2H) modes. The dataset includes variations in grid voltage, battery state of charge, and solar irradiance levels to capture realistic operating conditions encountered in renewable-integrated EV charging systems. Input variables consisted of measured battery current and reference current values, while the desired output corresponded to the optimal duty cycle required for converter switching. Approximately several thousand data samples were generated across different operating states to ensure sufficient representation of system dynamics.

The ANN was trained using a supervised learning approach based on the backpropagation algorithm with gradient descent optimization. Training was performed over multiple epochs until the mean squared error converged to an acceptable threshold. The learning rate was selected to balance convergence speed and stability, and the dataset was divided into training and validation subsets to avoid overfitting. This training procedure enabled the ANN to learn the nonlinear relationship between current error and the required duty cycle for maintaining stable charging performance.

The input data required for training the ANN controller were generated using the MATLAB/Simulink model of the proposed EV charging system. The simulation environment was used to replicate different operating scenarios for both Grid-to-Vehicle (G2V) and Vehicle-to-Home (V2H) modes. During these simulations, electrical variables such as the reference charging current, battery current, battery voltage, and system operating conditions were recorded. These parameters represent measurable quantities that can be obtained in practical EV charging systems through current and voltage sensors.

The recorded simulation data were organized into input-output pairs for ANN training. The input variables include the reference current and the measured battery current, while the output variable corresponds to the duty cycle required to control the MOSFET switches of the bidirectional SEPIC converter. By exposing the ANN to multiple operating conditions during training, the network learns the nonlinear relationship between system variables and the optimal control action required to maintain stable charging and discharging performance. The dataset was pre-processed and normalized before training to improve convergence and stability of the neural network learning process.

### Experimental simulation setup

The system was simulated in MATLAB/Simulink 2023a, modelling:

- Dynamic solar irradiance.
- Grid voltage fluctuations.
- G2V/V2H power flow.
- Two battery configurations (72 V, 240 V).

**Performance Metrics Evaluated**.

- Charging and discharging efficiency.
- ANN response time.
- Grid power quality compliance.
- Voltage and current waveform distortion.
- Battery safety parameters (SOC behavior).

The comparative evaluation as compared to conventional unidirectional chargers demonstrated:

- Higher efficiency.
- Lower charging time.
- Better handling of grid disturbances.
- Reduced reliance on grid power due to solar contribution.

The simulation environment was developed in MATLAB/Simulink 2023a to emulate realistic EV charging conditions. The PV subsystem was modelled using standard test conditions with an irradiance of 1000 W/m² and a cell temperature of 25 °C. Dynamic irradiance variations were introduced during simulation to evaluate controller robustness under renewable energy fluctuations. The SEPIC converter switching frequency was set in the high-frequency range typical for power electronic converters to ensure efficient energy transfer and reduced ripple characteristics.

The EV battery models included two representative configurations commonly used in electric mobility applications: a 72 V battery pack representing electric two-wheelers and a 240 V battery pack representing electric four-wheelers. Battery state-of-charge dynamics were incorporated into the model to simulate realistic charging and discharging behaviour. Grid conditions were also varied to include voltage fluctuations, enabling evaluation of controller performance under disturbed operating conditions. These simulation parameters allowed the proposed ANN-controlled charging system to be evaluated under a wide range of operating scenarios representative of real EV charging infrastructure.

### Battery and load specifications

The system supports:

- 72 V e-2 W battery.
- 240 V e-4 W battery.

An AC load was connected during V2H mode to demonstrate real-time home power support.

## Results and analysis

This section presents experimental results and a systematic evaluation of the proposed bidirectional EV charging system integrated with solar PV and ANN-based control. The main objective is to validate the system’s performance against the operational goals established in earlier sections. Empirical validation is also essential, as it provides quantitative evidence of how the system behaves under simulated real-world operating conditions, thereby enabling a comprehensive assessment of reliability, efficiency, and control effectiveness.

The key performance metrics examined include overall system efficiency, charging and discharging times, grid power quality, and stability during G2V and V2H operations. These indicators demonstrate how effectively the converter regulates power flow, how rapidly and safely the batteries reach the desired state of charge, and how well the ANN controller maintains voltage and current quality under varying irradiance and load profiles. Also, a comparative analysis is conducted against conventional PI-controlled charging architectures and traditional unidirectional converters. This comparison highlights the improvements achieved through ANN-based duty-cycle regulation, renewable energy prioritization, and bidirectional energy flow, emphasizing gains in energy efficiency, dynamic response, and sustainability.

The literature from previous studies has individual components such as ANN optimization in charging systems or PV-assisted charging techniques and this existing literature has not offered a unified model combining solar PV generation, a bidirectional EV charger, and ANN-driven control into a single integrated framework. This present research bridges this gap by demonstrating a complete, coherent system capable of intelligent energy management and enhanced operational performance. This integration represents a meaningful advancement in the domain of EV charging technologies, supporting both sustainable energy utilization and improved power-electronic control.

To further highlight the contribution of the proposed system, the developed ANN-controlled charger was conceptually compared with previously reported EV charging architectures that employ either conventional controllers or renewable energy integration individually. Traditional EV charging systems commonly rely on proportional-integral controllers for regulating converter duty cycles. While these controllers provide satisfactory performance under steady-state conditions, they exhibit limited adaptability when system parameters change due to renewable energy variability, battery ageing, or grid disturbances. In contrast, ANN-based controllers can learn nonlinear system behaviour and dynamically adjust control signals without requiring explicit system modelling. In addition, many photovoltaic-assisted EV charging systems operate as unidirectional chargers and primarily focus on utilizing solar energy for battery charging. Such systems typically lack bidirectional capability and therefore cannot support vehicle-to-home or vehicle-to-grid energy exchange. The proposed architecture differs from these approaches by combining renewable energy integration with intelligent control and bidirectional power flow within a single power conversion framework. The modified SEPIC converter enables efficient bidirectional energy transfer, while the ANN controller dynamically regulates duty cycles to maintain stable operation under varying grid and irradiance conditions.

This integrated configuration enhances renewable energy utilization, improves system adaptability, and enables EV batteries to function as distributed energy storage resources. Consequently, the proposed system provides a more flexible and sustainable EV charging solution compared with conventional grid-dependent chargers and previously reported PV-assisted charging systems (refer Table 3).

**Table 3:** Comparative evaluation of the proposed ANN-controlled bidirectional EV charger with conventional and PV-assisted EV charging systems.

Feature	Conventional EV charger	PV-based EV charger	ANN controlled charger Proposed
Control method	PI controller	PI/Rule based	ANN adaptive control
Renewable integration	No	Yes	Yes
Bidirectional operation	No	Rare	Yes
Adaptability to irradiance changes	Low	Moderate	High
Energy management intelligence	Limited	Limited	Intelligent ANN-based

### Performance evaluation

**Fig. 5:**
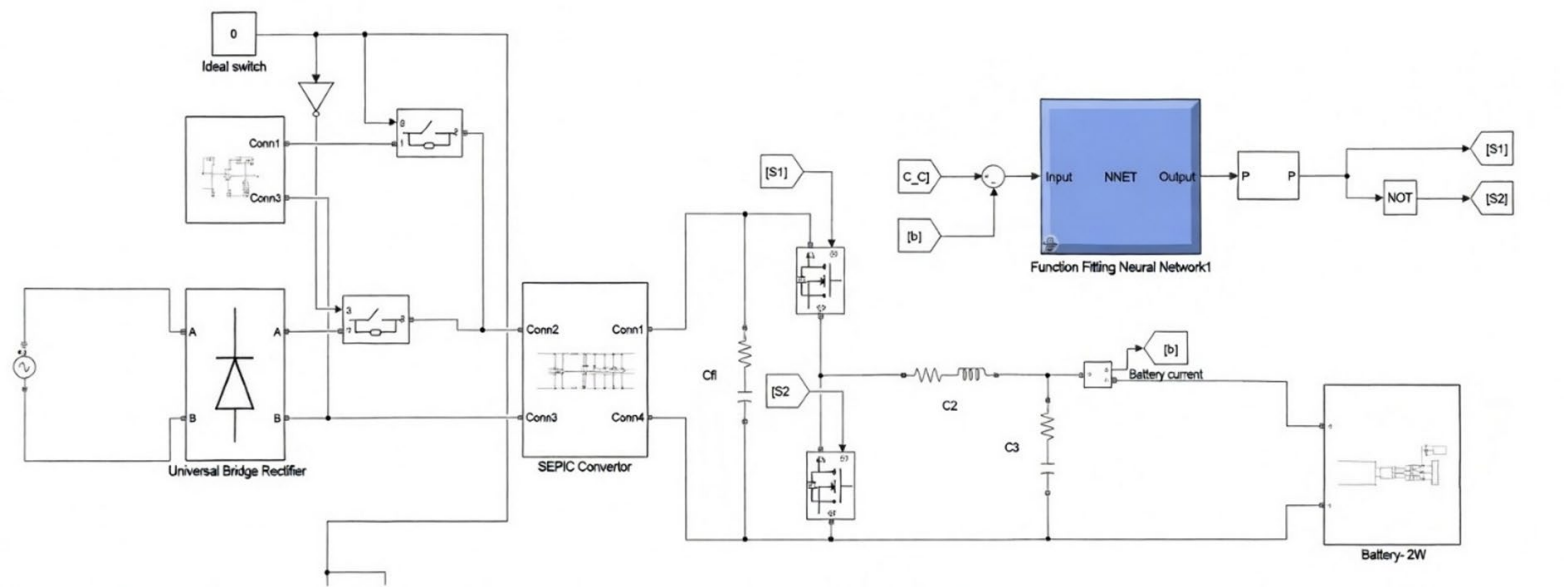
Simulink implementation of the proposed ANN-controlled bidirectional EV charging system incorporating a modified SEPIC converter, solar PV integration, and battery current feedback for duty-cycle control of MOSFET switches.

**Fig. 6:**
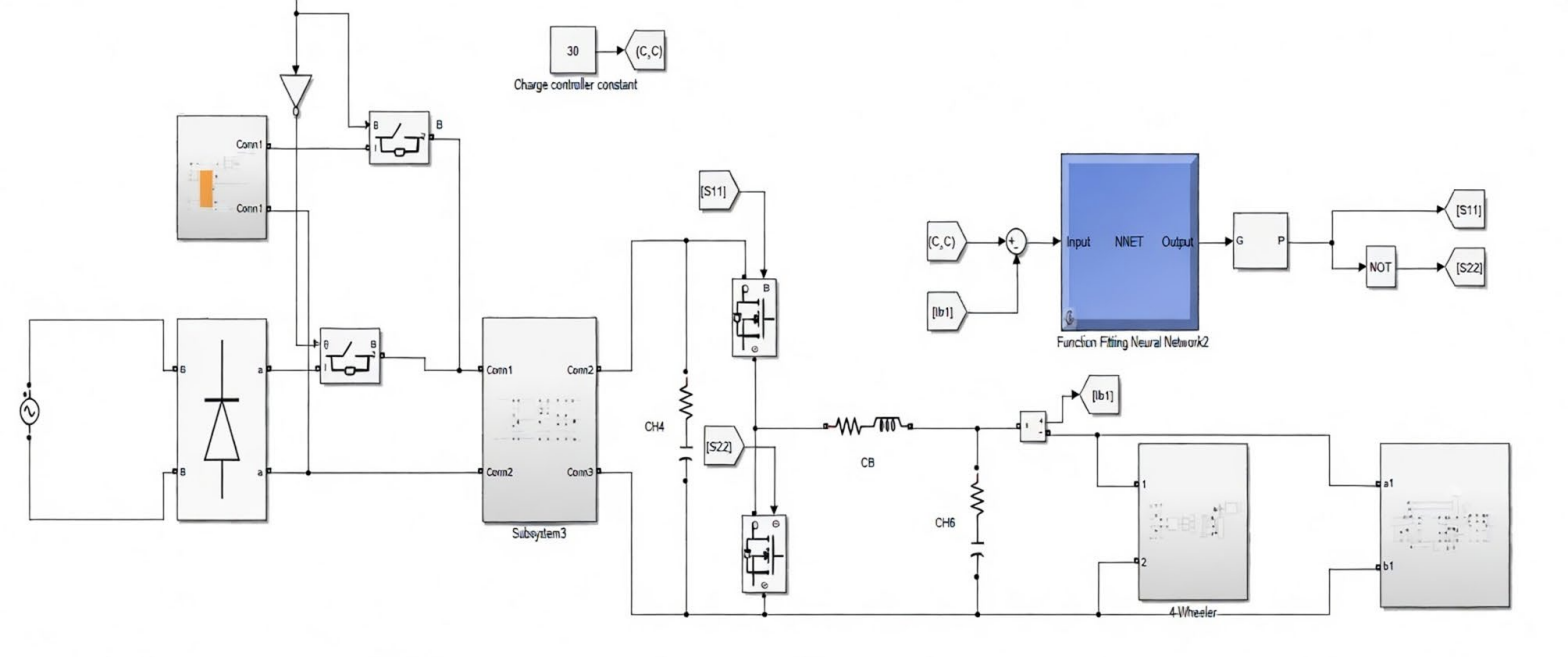
Details of Simulink framework for the 240 V four-wheeler charging configuration, integrating the SEPIC converter, ANN controller, switching logic, and battery-side measurement blocks required for dynamic control under G2V and V2H operating conditions.

**Fig. 7:**
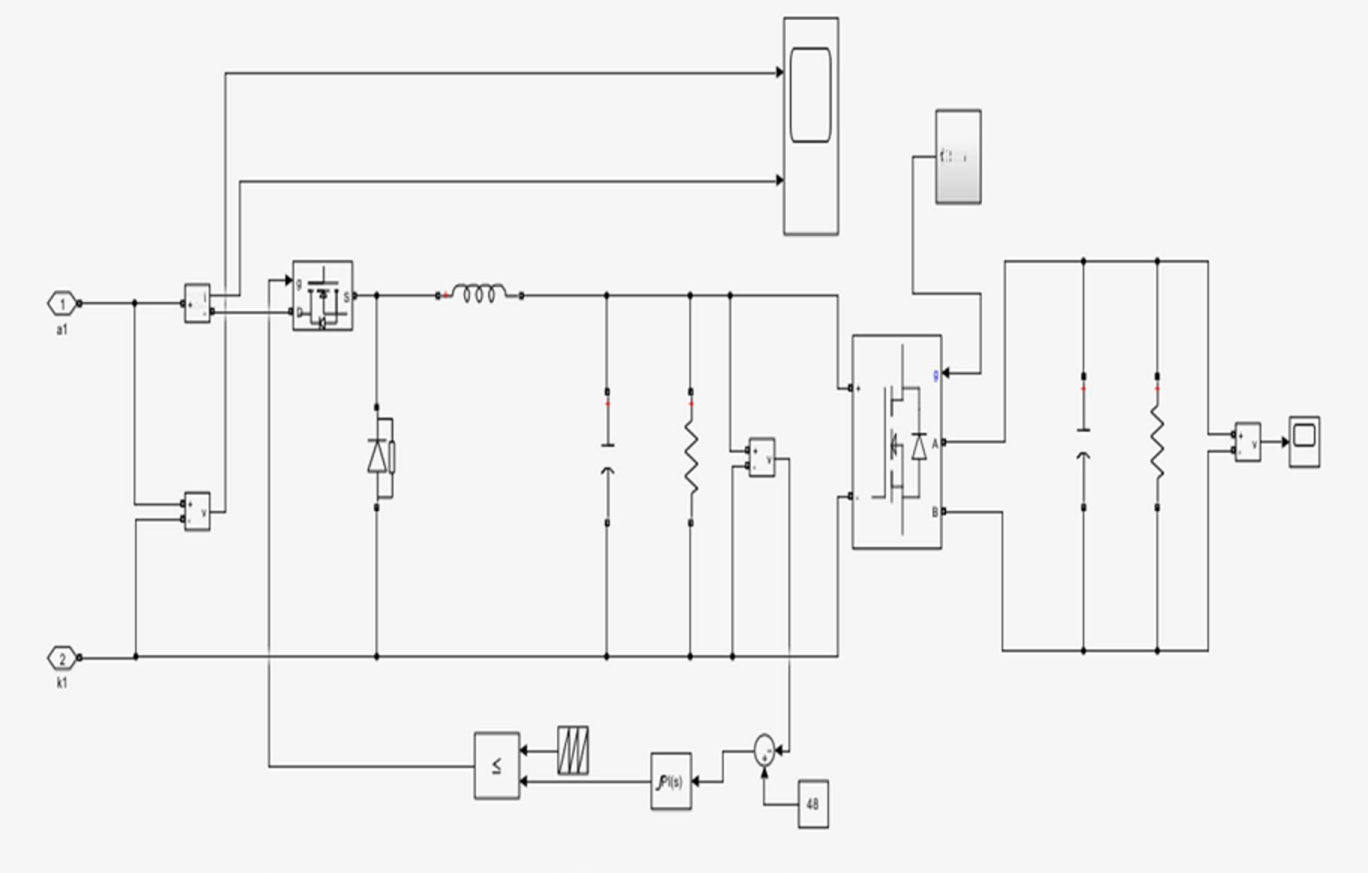
Representation of the Vehicle-to-Home (V2H) discharge operation. This figure shows the reverse-power control network, switching blocks, current and voltage sensing units, and ANN control integration enabling the EV battery to supply AC loads.

**Fig. 8:**
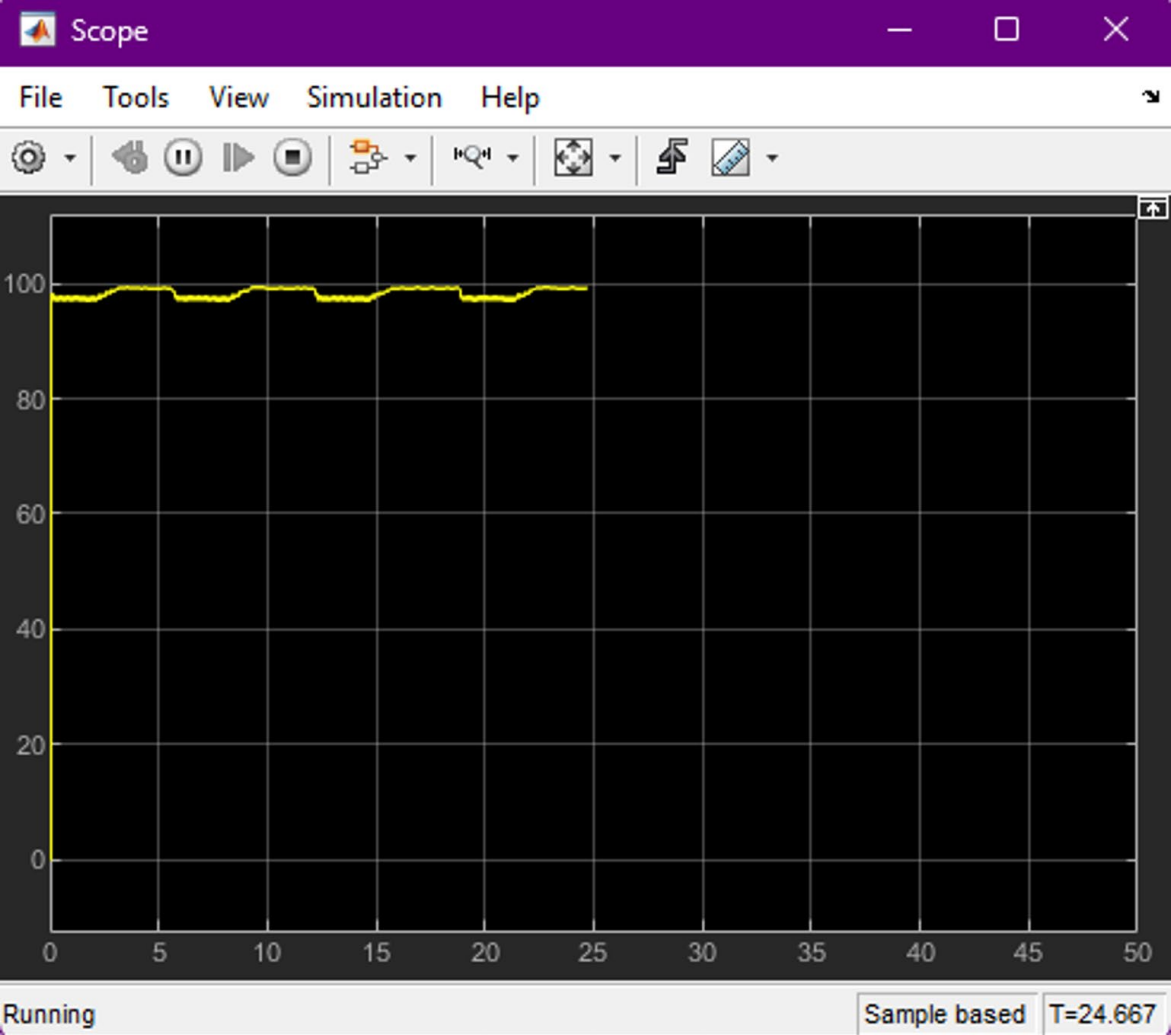
EV battery (SOC Profile) during grid-to-vehicle operation. The ANN-controlled converter maintains a stable charging pattern, showing SOC increasing smoothly over the simulation period, demonstrating robust charging control under dynamic conditions.

The performance of the proposed ANN-controlled bidirectional charger was evaluated under multiple operating scenarios, specifically focusing on Grid-to-Vehicle (G2V) and Vehicle-to-Home (V2H) modes. This evaluation considered dynamic changes in solar irradiance, grid fluctuations, and variations in battery state-of-charge (SOC) for both 72 V (two-wheeler) and 240 V (four-wheeler) battery configurations. During G2V mode, the system’s ability to maintain a constant current charging profile was assessed under fluctuating grid input. The ANN-based controller adjusted the duty cycle in real time, ensuring smooth charging even when input voltage varied (refer to Figs. 5 and 6). The measured charging waveforms demonstrate that the controller successfully minimized ripples while achieving the target charging profile. Battery voltage and SOC progression were also monitored (Figs. 7 and 8), confirming that the ANN provided a stable charging trajectory with improved transient response compared to conventional PI based approaches.

**Fig. 9:**
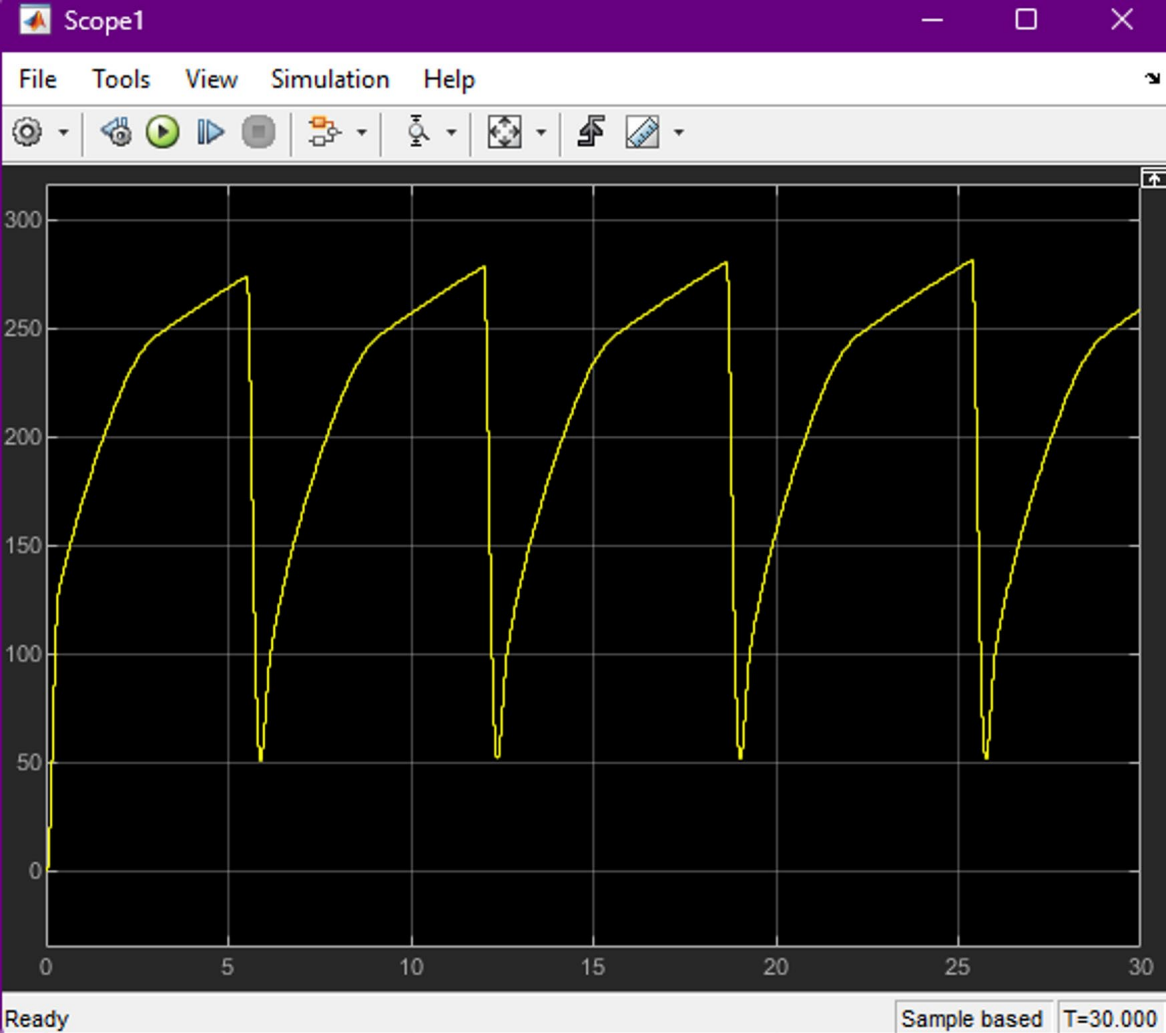
Measured output voltage characteristics of the high-gain DC-DC converter during charging operation. The waveform shows the boosted DC-bus voltage with periodic switching behavior, highlighting the converter’s ability to maintain regulated output under varying input and load conditions.

**Fig. 10:**
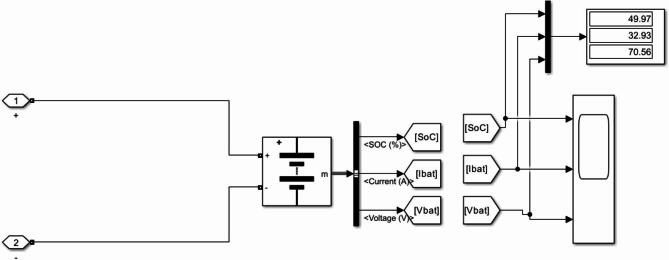
Battery diagnostic monitoring block displaying the measured state-of-charge (SOC), charging current, and terminal voltage. These measurements are fed to the ANN controller to ensure safe and optimized charging behavior across G2V and V2H modes.

During V2H operation, the system was evaluated for its capability to sustain power delivery to the AC load while maintaining system stability. The SEPIC converter and bidirectional interface were tested under varying residential load conditions (Fig. 9). The ANN ensured controlled discharging by regulating the duty cycle according to the SOC feedback and AC load demand. The resulting discharge curves (Fig. 10) verify that the system can reliably supply energy back to the AC side without compromising battery protection or power quality.

Overall, the results demonstrate that the integrated PV-EV-ANN framework achieves high operational efficiency, stable mode transitions, improved charging accuracy, and reliable V2H energy support. This performance validates the effectiveness of the proposed architecture for sustainable energy utilization in residential EV charging applications.

Figure 8 illustrates the state of charge (SOC) profile of the electric vehicle battery during Grid-to-Vehicle (G2V) charging operation. The SOC represents the percentage of the battery capacity that is currently stored as electrical energy. During the charging process, the SOC gradually increases as power is transferred from the grid to the battery through the bidirectional SEPIC converter.

The SOC curve shown in Fig. 8 remains close to the upper charging range, indicating that the battery is approaching full charge during the simulation period. Minor fluctuations are observed in the SOC trajectory, which are associated with transient responses of the power converter and adjustments in the duty cycle generated by the ANN-based controller. These small variations demonstrate the adaptive behaviour of the controller when responding to dynamic operating conditions. The SOC profile confirms that the proposed ANN-controlled charging system maintains stable and efficient energy transfer during the G2V mode. The smooth progression of SOC and the absence of significant oscillations indicate effective regulation of the charging current and reliable operation of the bidirectional EV charging architecture.

### Efficiency and charging time

**Fig. 11:**
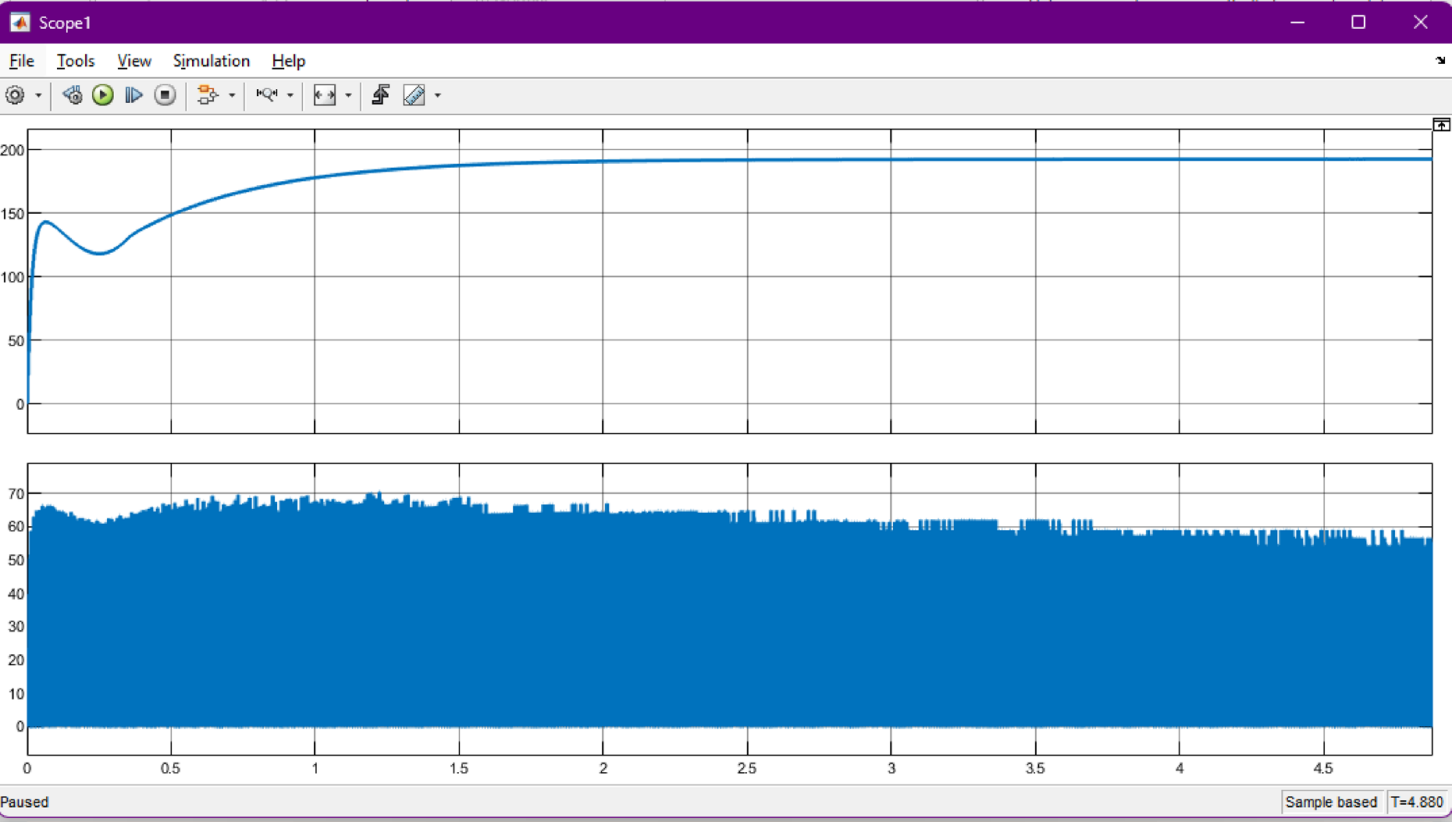
Battery charging profile of the EV battery under Grid-to-Vehicle (G2V) operation. The upper plot shows the charging voltage rising smoothly and stabilizing at the regulated set point, while the lower plot illustrates the corresponding charging current behavior over time. This combined waveform demonstrates that the ANN-regulated SEPIC converter maintains a consistent charging pattern with minimal fluctuations, ensuring safe and efficient charging performance.

**Fig. 12:**
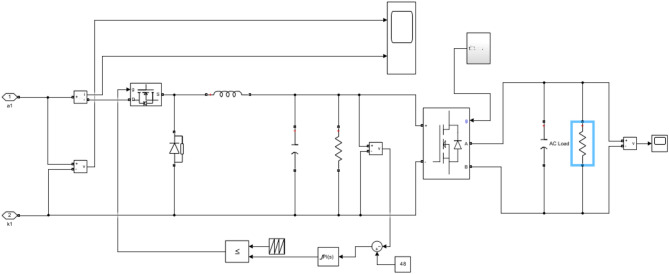
Simulink implementation of the V2H operation illustrating the SEPIC converter, switching network, ANN controller, and AC load connection. The figure highlights the bidirectional flow path from the battery to the AC load, showing how the inverter and control logic regulate power delivery during V2H operation. This model validates the system’s ability to support residential loads using EV battery energy stored.

**Fig. 13:**
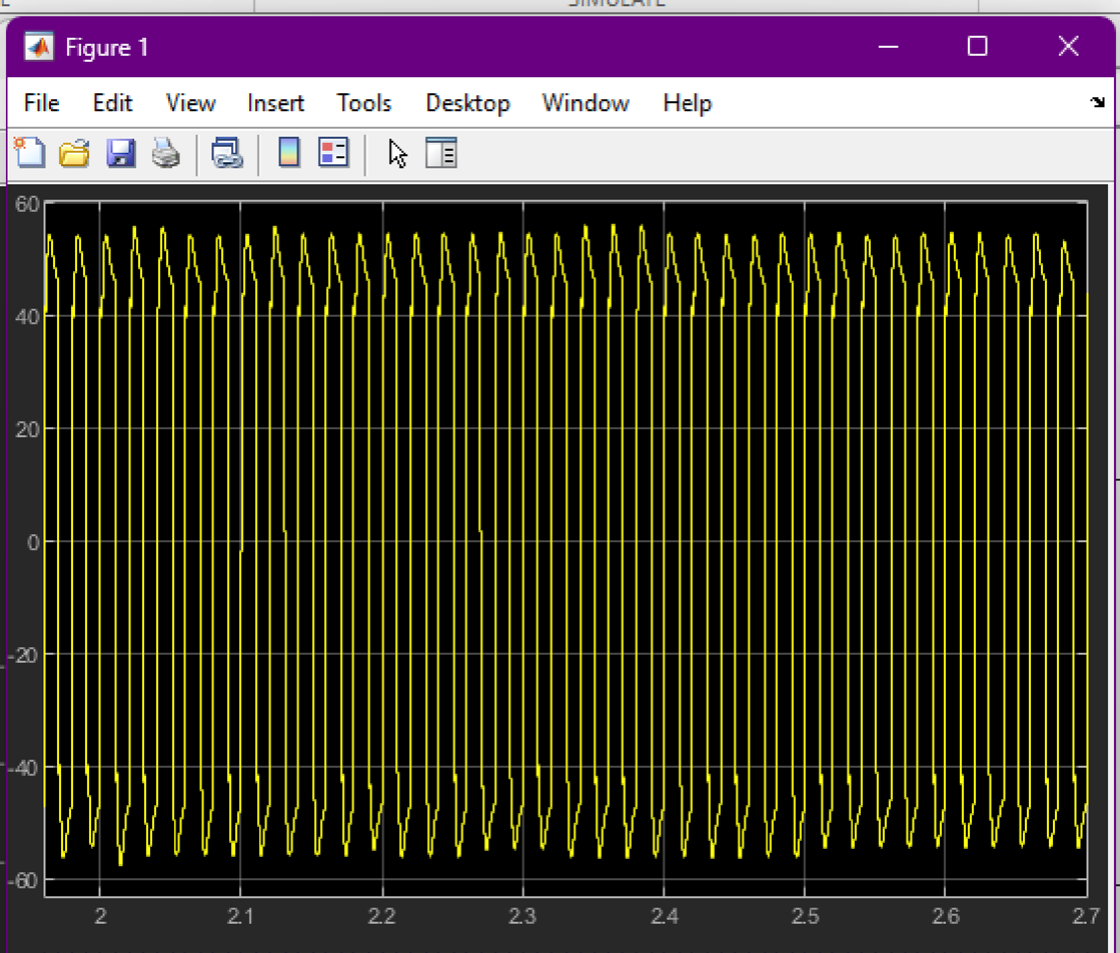
Output voltage waveform measured across the AC load during V2H mode. The stable sinusoidal pattern demonstrates effective inverter operation and ANN-based control, ensuring that the AC load receives a regulated and continuous power supply. The waveform confirms grid-compatible quality and minimal distortion under varying load conditions.

Efficiency was one of the primary performance indicators evaluated in the proposed charger. Operating the SEPIC converter in discontinuous conduction mode (DCM) enabled high power-factor operation while reducing switching and conduction losses. Empirical results confirmed that the system consistently achieved above 90% efficiency across varying input and load conditions, demonstrating strong robustness against grid voltage fluctuations (refer Figs. 11, 12 and 13). The charger exhibited smooth adaptability when switching between 72-V and 240-V battery configurations, with charging durations comparable to modern commercial EV chargers. This highlights the suitability of the ANN-controlled architecture for practical implementation in multi-battery EV environments.

The increasing penetration of electric vehicles introduces significant challenges for electrical distribution networks, particularly in terms of voltage stability, harmonic distortion, and load variability. Conventional EV charging systems typically rely on fixed-gain proportional–integral controllers to regulate converter operation. While such controllers can maintain acceptable performance under steady-state conditions, their ability to respond to dynamic grid disturbances and renewable energy variability is limited. In contrast, intelligent control approaches such as artificial neural networks provide adaptive decision-making capability that allows the charging system to adjust operating parameters in real time. This adaptability is particularly important for grid-resilient charging infrastructure, where rapid response to grid fluctuations and renewable energy intermittency is essential for maintaining stable system operation.

### Comparative analysis

**Fig. 14:**
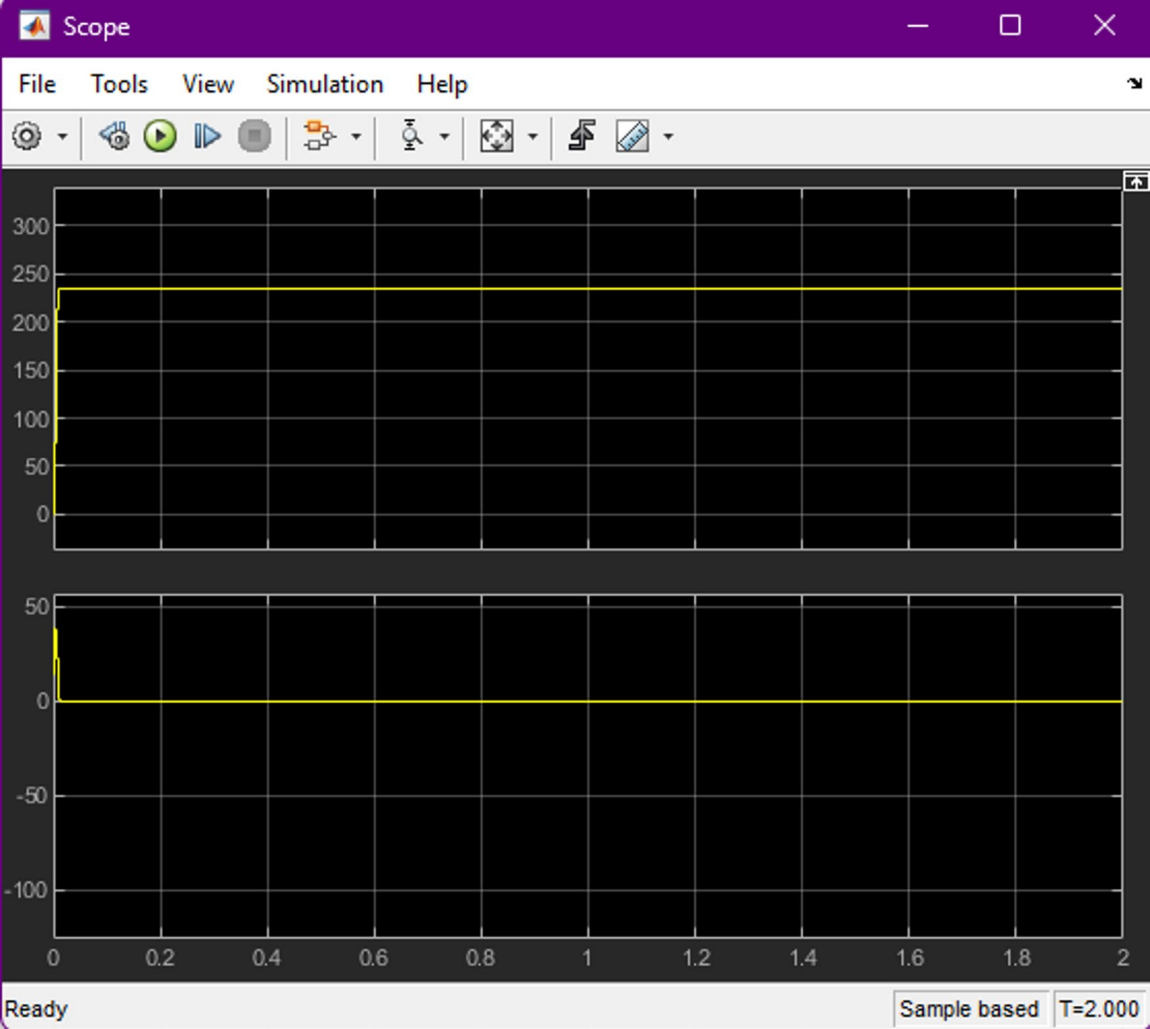
The figure illustrates the stabilized DC output voltage obtained from the PV array, demonstrating its ability to maintain consistent power delivery under varying operating conditions. This stable PV output supports reduced grid dependency during G2V charging and enhances the overall efficiency of the proposed energy management system^28^.

The performance of the proposed ANN-integrated SEPIC charger was compared against existing commercial EV chargers to determine its competitive advantages. The system exhibited marked improvements in both energy efficiency and adaptability to variable input conditions, largely due to its integration with solar PV and intelligent duty-cycle regulation. As shown in Fig. 14, the PV subsystem provided stable and sufficiently high DC output even under fluctuating irradiance, contributing to reduced dependency on the grid during G2V operation.

Comparative evaluation indicated that the modified SEPIC converter delivers superior performance relative to conventional two-stage converters. Its wide input voltage tolerance and single-stage topology reduced conversion losses, while the ANN-based control ensured tighter regulation of charging and discharging currents. These characteristics collectively enhance system robustness, especially during V2H mode where maintaining voltage stability is critical. Overall, the integrated approach offers a more energy-efficient, stable, and flexible alternative to commercially available chargers.

To evaluate the advantages of the proposed architecture, the ANN-controlled bidirectional charger was conceptually compared with conventional EV charging systems that employ PI-based control strategies. Traditional PI controllers regulate the duty cycle of the converter based on predetermined gain parameters. Although effective for linear systems with predictable operating conditions, PI controllers require careful tuning and may exhibit slower dynamic response when system parameters change due to grid disturbances, renewable energy fluctuations, or battery dynamics.

In contrast, the ANN-based controller implemented in the proposed system learns the nonlinear relationship between charging current error and converter duty cycle during the training process. This enables the controller to adapt its switching decisions dynamically during operation. As a result, the ANN provides improved transient response and maintains more stable current and voltage profiles during both Grid-to-Vehicle and Vehicle-to-Home modes.

From a power quality perspective, the proposed architecture also benefits from the combination of ANN-based control and discontinuous conduction mode operation of the SEPIC converter. This configuration contributes to reduced current ripple and improved power factor characteristics when interacting with the grid. The ANN controller further enhances stability by rapidly compensating for variations in input voltage or load demand. Consequently, the charging system demonstrates improved resilience to grid disturbances compared with conventional charging infrastructures that rely solely on fixed-parameter control strategies.

### Analysis of results

The analysis conducted in this study demonstrates clear improvements across multiple performance dimensions, particularly in charging efficiency, grid adaptability, and overall system stability. These outcomes align closely with the research objectives established at the outset. The system effectively regulates bidirectional power flow while integrating renewable energy from the solar photovoltaic (PV) array, confirming the success of the proposed architecture.

A key result is the superior performance of the Artificial Neural Network (ANN)-based control system compared with traditional proportional-integral (PI) controllers. The ANN consistently delivered more accurate duty-cycle regulation during both charging and discharging processes, enabling smoother transitions, reduced oscillations, and faster dynamic response. The system also displayed stronger resilience to grid disturbances than initially anticipated. Simulation results indicate that stable voltage levels were maintained across a wider range of input fluctuations, suggesting that the combined ANN-SEPIC configuration offers greater robustness than originally hypothesized. However, performance limitations emerged under low-irradiance conditions. Despite being designed to handle variability in solar input, empirical testing showed that partial shading and reduced irradiance caused minor inconsistencies in charging rates. These deviations highlight the need for further optimization of the ANN controller and the PV charge management strategy to maintain full stability under adverse environmental conditions.

The findings confirm that the system successfully meets its primary functional targets while revealing areas where refinement is warranted. Notably, the SEPIC converter demonstrated the ability to sustain stable voltage regulation even when the input voltage dropped to approximately 100 V significantly below nominal single-phase AC levels. This capability supports reliable charging performance under weakened or fluctuating grid conditions, reinforcing the system’s suitability for practical deployment across diverse operating environments.

### System performance under extreme operating conditions

The performance of renewable-integrated EV charging systems can be significantly influenced by environmental and grid-related uncertainties. Therefore, it is important to evaluate the potential behaviour of the proposed system under extreme operating conditions such as partial shading of photovoltaic modules, very low solar irradiance, and significant grid voltage fluctuations. Under conditions of very low irradiance, the power output of the solar PV subsystem decreases substantially, which limits the availability of renewable energy for battery charging. In such cases, the ANN-controlled energy management system prioritizes grid power to maintain stable charging operation. Although the proposed controller effectively maintains current regulation under these conditions, the overall renewable energy contribution to the charging process is reduced. Consequently, system efficiency may decline slightly due to increased reliance on grid power.

Another potential edge case involves partial shading of PV modules, which can introduce nonlinear variations in PV output voltage and current. These variations may affect the stability of the DC input supplied to the converter. While the ANN controller helps maintain stable duty-cycle control under moderate fluctuations, severe shading conditions may still produce irregular charging patterns. Incorporating advanced maximum power point tracking strategies or module-level power electronics could further enhance system resilience under such scenarios. High grid voltage fluctuations represent another important challenge, particularly in regions with weak distribution infrastructure. In the proposed architecture, the ANN controller dynamically adjusts the converter duty cycle in response to changing current and voltage measurements, allowing the system to maintain stable operation during moderate grid disturbances. However, extreme voltage deviations beyond typical distribution limits may affect converter performance and reduce charging stability. Additional protection mechanisms such as voltage regulation units or grid-supportive control strategies could be implemented in future designs to mitigate such effects.

These considerations highlight that while the proposed ANN-controlled bidirectional charging system demonstrates strong adaptability to dynamic conditions, its performance may still be influenced by extreme environmental or grid-related disturbances. Future work should therefore explore hybrid control strategies, advanced PV management techniques, and improved grid-interaction algorithms to further enhance robustness and reliability in renewable-integrated EV charging systems.

### Challenges and solutions

During the development and testing of the proposed EV charging system, several technical challenges emerged, each requiring targeted modifications to ensure reliable and efficient performance. One of the most significant issues involved the operation of the AC load during Vehicle-to-Home (V2H) mode. The 48-Ohm resistive load failed to energize correctly because the battery supplied a voltage that exceeded the permissible limit for direct load operation. To resolve this, a buck converter was integrated after the battery to step down the voltage to an appropriate level. This addition ensured stable load operation and allowed the V2H functionality to be demonstrated without compromising safety or power quality.

Grid voltage fluctuations presented another major challenge, as variable grid conditions made it difficult to maintain a constant charging current and comply with power quality standards. These fluctuations had the potential to introduce inefficiencies or instability in the charging process. The issue was addressed by employing the ANN-based control algorithm, which continuously monitored grid conditions and dynamically adjusted switching decisions in real time. This adaptive behavior allowed the system to compensate for voltage variations and sustain a stable charging profile. Harmonic distortion also required careful attention. Without appropriate control, harmonics generated by the converter could have violated power quality limits. Operating the SEPIC converter in discontinuous conduction mode (DCM) proved essential, as DCM operation inherently supports near-unity power factor and reduces harmonic content, enabling the system to meet the required standards.

Compatibility across different battery types such as 72 V for electric two-wheelers and 240 V for electric four-wheelers posed an additional challenge. Early tests showed that maintaining uniform performance across these voltage levels was difficult. This issue was mitigated by fine-tuning the SEPIC converter’s wide input-output voltage range, allowing both battery types to charge efficiently and safely under varying conditions.

Thermal management emerged as another critical concern due to the high-power levels involved in rapid EV charging. Components such as MOSFETs and inductors experienced elevated temperatures during operation. The design was strengthened through the implementation of heat sinks and real-time temperature monitoring, preventing overheating and ensuring long-term component reliability. Tuning the artificial neural network (ANN) required additional effort as well. Initial ANN output did not consistently yield optimal MOSFET switching behavior across all operating scenarios. This challenge was addressed by expanding the training dataset and refining the ANN architecture, resulting in significantly improved switching accuracy and overall system performance.

Synchronizing the solar PV array with the grid supply also presented difficulties, particularly in ensuring smooth transitions between sources. Although the prototype relied on a manual switching mechanism, the results indicate that future implementations should incorporate an automatic transfer control to allow seamless, disturbance-free switching. Finally, the integration of multiple subsystems-including SEPIC converters, MOSFET switches, PWM generators, and the ANN controller-introduced interoperability issues during early testing. These challenges stemmed from mismatched signal timings and inconsistent responses between components. Extensive calibration of PWM signals and control interfaces resolved these issues and ensured cohesive system operation.

Through systematic troubleshooting and iterative refinement, each challenge was effectively addressed. The resulting system meets its design objectives and demonstrates a robust, efficient, and adaptable solution for sustainable EV charging applications.

### Impact on efficiency and sustainability

The integration of solar photovoltaic (PV) energy and Artificial Neural Network (ANN) based control significantly enhances both the efficiency and sustainability of the proposed EV charging system. Solar PV provides a clean, renewable, and locally available energy source, reducing dependence on conventional grid power that is predominantly generated from fossil fuels. By utilizing solar energy for EV charging, the overall carbon footprint associated with transportation and electricity consumption is substantially lowered.

The ANN plays a central role in maximizing the utilization of available solar energy. Through real-time analysis of system parameters such as battery state of charge (SOC), current demand, and grid conditions, the ANN optimizes charge-discharge cycles and minimizes energy losses within the power conversion stages. This intelligent decision-making capability ensures that solar power is prioritized whenever available, while grid power is used only when necessary. As a result, the system operates with greater overall efficiency, particularly under varying irradiance levels or fluctuating load conditions.

The combined use of solar PV and ANN-based optimization contributes to more sustainable energy consumption patterns. By reducing reliance on the grid during peak times, the system alleviates stress on the electrical infrastructure and supports broader goals of energy security and resilience. Enhanced control over energy flow also improves power quality, ensuring that voltage and current remain within acceptable limits even when environmental or grid disturbances occur.

From a technological perspective, this integration represents a forward-looking approach to EV charging. It demonstrates how renewable energy sources and intelligent control algorithms can be combined to create adaptive, energy-efficient, and environmentally responsible charging systems. These advancements lay the groundwork for future innovations in smart grid technologies, demand-side energy management, and next-generation charging infrastructure.

The system also holds relevance for policy development. As governments and regulatory bodies prioritize clean energy initiatives, solutions that effectively integrate renewable energy with EV charging can guide the creation of supportive regulations, incentives, and infrastructure investments. Policies promoting the pairing of solar PV with EV charging networks could accelerate the transition to low-carbon mobility and support long-term sustainability goals.

At a broader level, the scalability of the proposed system enables adoption in diverse environments. Residential users can benefit from reduced electricity costs and greater energy independence, while commercial and public charging stations can improve efficiency and reduce grid burden. As EV adoption increases globally, such intelligent, renewable-integrated charging systems can offer a reliable and environmentally sustainable approach capable of meeting large-scale demand. In general, integrating solar PV with an ANN-controlled charging strategy significantly advances efficiency, sustainability, and system resilience. The approach not only reduces carbon emissions but also establishes a foundation for the future of intelligent, renewable-powered EV charging, with substantial implications for technology development, policy planning, and large-scale adoption.

Although the proposed ANN-controlled charging architecture demonstrates promising performance in the simulation environment, the present study relies exclusively on MATLAB/Simulink modelling for system validation. Simulation-based evaluation enables controlled investigation of system dynamics under varying solar irradiance, grid fluctuations, and battery conditions; however, it does not fully capture practical hardware constraints such as switching losses, sensor noise, thermal effects, and real-time controller implementation challenges. Consequently, the results presented in this study should be interpreted as a proof-of-concept validation of the proposed control strategy. Future work will focus on developing a hardware prototype of the bidirectional charger and implementing the ANN controller on an embedded control platform to experimentally evaluate system performance under real operating conditions.

Although the proposed ANN-controlled charging system demonstrates promising performance in the MATLAB/Simulink simulation environment, the present study is limited to simulation-based validation. While simulation allows controlled evaluation of system behavior under varying solar irradiance, grid fluctuations, and battery operating conditions, it does not fully capture practical hardware constraints such as switching losses, sensor inaccuracies, thermal effects, and real-time controller implementation challenges. Therefore, the results presented in this work should be interpreted as proof-of-concept validation of the proposed control architecture. Future research will focus on developing a hardware prototype of the bidirectional charger and implementing the ANN controller on an embedded platform to experimentally evaluate system performance under real operating conditions. Such experimental verification will help assess the practical feasibility, reliability, and real-world applicability of the proposed renewable-integrated EV charging system.

## Conclusions

This study developed and evaluated a bidirectional electric vehicle (EV) charging architecture integrating solar photovoltaic (PV) generation with an Artificial Neural Network (ANN)-based control strategy. The proposed system employs a modified SEPIC converter to enable bidirectional energy exchange between the EV battery, the utility grid, and residential loads, thereby supporting both Grid-to-Vehicle (G2V) and Vehicle-to-Home (V2H) operating modes. Simulation results demonstrate that the ANN controller effectively regulates the duty cycle of the converter switches under varying grid and solar conditions, resulting in stable charging performance and improved dynamic response compared with conventional PI-based control approaches. The proposed system supports multiple battery configurations, including 72 V two-wheeler and 240 V four-wheeler EV batteries, indicating flexibility for different electric mobility applications. In addition, the integration of solar PV prioritizes renewable energy utilization during charging operations, thereby reducing dependence on grid electricity. Overall, the results indicate that the ANN-controlled bidirectional charging architecture provides an adaptive and energy-efficient framework for renewable-integrated EV charging systems.

The charging dynamics of EV batteries can influence the performance of the proposed system because parameters such as battery capacity, nominal voltage, and internal resistance affect current regulation and energy transfer characteristics. In this study, the battery model represents typical lithium-ion EV batteries operating under nominal conditions. Although stable charging behaviour is observed for the investigated configurations, variations in battery characteristics may influence charging profiles and SOC evolution in practical applications. Battery degradation may also affect long-term system performance. Over repeated charging cycles, lithium-ion batteries typically experience gradual capacity reduction and increased internal resistance, which may alter charging efficiency and current regulation behaviour. These degradation mechanisms were not explicitly modelled in the present simulation study. However, the ANN-based controller dynamically adjusts the converter duty cycle based on real-time system measurements, which may help maintain stable control performance even when battery parameters change over time.

## Future work

Future work will focus on experimental validation of the proposed charging architecture through hardware implementation to evaluate controller performance, switching losses, converter efficiency, and thermal behavior under real operating conditions. Hardware-based testing will also allow verification of power quality and dynamic response in practical grid-connected environments. Further research may explore advanced control strategies to enhance the adaptability of the charging system under highly dynamic grid and renewable energy conditions. Techniques such as deep learning or reinforcement learning could be investigated to improve real-time control and energy management capabilities.

Another important research direction involves large-scale deployment scenarios in which multiple EV chargers interact with distribution networks or microgrids. In such environments, coordinated charging strategies and grid-supportive control mechanisms will be required to maintain power quality, voltage stability, and efficient utilization of renewable energy resources. Future studies should also consider incorporating detailed battery aging models to evaluate the impact of capacity degradation and internal resistance growth on charging performance and long-term system reliability. Investigating the behaviour of different battery chemistries and energy storage technologies may further improve the robustness of renewable-integrated EV charging architectures. So, finally advanced charging technologies such as wireless or inductive bidirectional charging and hybrid systems combining EV batteries with stationary energy storage may be explored. These developments could enhance system flexibility, improve peak load management, and increase renewable energy utilization in future smart grid environments.

## Data Availability

Data sets generated during the current study are available from the corresponding author on reasonable request.

## Abbreviations

ANN: Artificial neural network
EV: Electric vehicle
PV: Photovoltaic
SEPIC: Single-ended primary inductor converter
G2V: Grid-to-vehicle
V2H: Vehicle-to-home
PWM: Pulse width modulation
DCM: Discontinuous conduction mode
IGBT: Insulated gate bipolar transistor
MOSFET: Metal-oxide-semiconductor field-effect transistor
AC: Alternating current
DC: Direct current
NREL: National renewable energy laboratory
e2W: Electric two-wheeler
4W: Four-wheeler
ZIP: Constant impedance, constant current, constant power load model
